# Medicinal Potentialities of Plant Defensins: A Review with Applied Perspectives

**DOI:** 10.3390/medicines6010029

**Published:** 2019-02-19

**Authors:** Nida Ishaq, Muhammad Bilal, Hafiz M.N. Iqbal

**Affiliations:** 1 School of Agriculture and Biology, Shanghai Jiao Tong University, Shanghai 200240, China; nidaishaq88@yahoo.com; 2 School of Life Science and Food Engineering, Huaiyin Institute of Technology, Huaian 223003, China; 3 Tecnologico de Monterrey, School of Engineering and Sciences, Campus Monterrey, Ave. Eugenio Garza Sada 2501, CP 64849 Monterrey, N.L., Mexico

**Keywords:** defensins, secondary metabolites, plant defense, antimicrobial and anticancer activity, medicine, innate immunity

## Abstract

Plant-based secondary metabolites with medicinal potentialities such as defensins are small, cysteine-rich peptides that represent an imperative aspect of the inherent defense system. Plant defensins possess broad-spectrum biological activities, e.g., bactericidal and insecticidal actions, as well as antifungal, antiviral, and anticancer activities. The unique structural and functional attributes provide a nonspecific and versatile means of combating a variety of microbial pathogens, i.e., fungi, bacteria, protozoa, and enveloped viruses. Some defensins in plants involved in other functions include the development of metal tolerance and the role in sexual reproduction, while most of the defensins make up the innate immune system of the plants. Defensins are structurally and functionally linked and have been characterized in various eukaryotic microorganisms, mammals, plants, gulls, teleost species of fish, mollusks, insect pests, arachnidan, and crustaceans. This defense mechanism has been improved biotechnologically as it helps to protect plants from fungal attacks in genetically modified organisms (GMO). Herein, we review plant defensins as secondary metabolites with medicinal potentialities. The first half of the review elaborates the origin, structural variations, and mechanism of actions of plant defensins. In the second part, the role of defensins in plant defense, stress response, and reproduction are discussed with suitable examples. Lastly, the biological applications of plant defensins as potential antimicrobial and anticancer agents are also deliberated. In summary, plant defensins may open a new prospect in medicine, human health, and agriculture.

## 1. Introduction

In nature, plants are continuously confronted with attacks from pests and other microbial pathogens such as fungi and bacteria. In addition, plants also suffer and face harsh environmental conditions of salt and drought stress. To overcome these stresses, plants have established a very complex mechanism to protect themselves from pests, pathogens, and fungal attacks [1]. Besides, many other defense factors including polyacetylenes, phenolics, alkaloids, terpenoids, and hydrogen peroxide are also generated to circumvent these kinds of occurrences. Along with the above-described chemicals, plants also released an array of defensins and defensin-related proteins [2,3].

**Figure 1:**
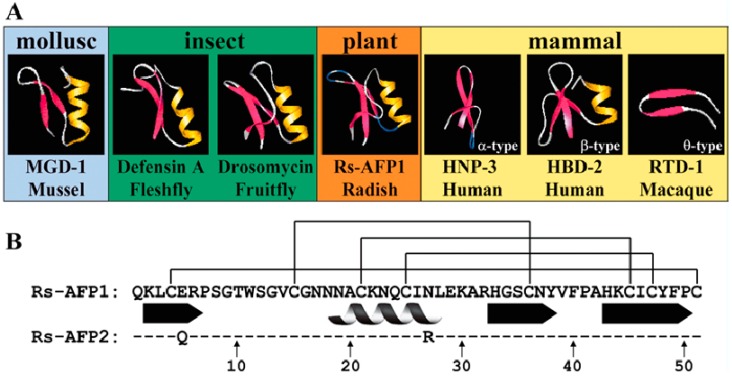
(**A**) Three-dimensional structure of defensins of plant, invertebrate (insect and mollusk), and vertebrate (mammalian) origin. Structures were downloaded from the protein data bank (http://www.rcsb.org/pdb; PDB accession ID numbers: MGD-1: 1FJN, defensin A: 1ICA, drosomycin: 1MYN, Rs-AFP1: 1AYJ, HNP-3: 1DFN, HBD-2: 1FD3, RTD-1: 1HVZ). Pictures were generated using Rasmol software. The α-helices and β-sheets are shown in yellow and red, respectively. (**B**) The amino acid sequence of mature Rs-AFP1 and 2. Dashes indicate identical amino acid residues. Connecting lines between cysteine residues represent disulfide bonds, while the spiral and arrows indicate the location of the α-helix and β-strands, respectively. Adapted from Thomma et al. [5], with permission from Springer Nature. Copyright (2002) Springer-Verlag.

Plant defensins are cysteine-rich highly stable peptides of 4–45 amino acid residues, comprising a part of the immune system that can present antifungal, antibacterial, or proteinase inhibitory activity. These peptides display a conserved three-dimensional structure containing α-helix and triple-stranded β-sheet stabilized into a compact structure through disulfide linkages [4]. This structure resembles defense peptides in insects and mammals, revealing a common historic origin. Moreover, only one class, namely defensins, seems to be conserved between invertebrates, plants, and vertebrates (Figure 1) [5]. Nowadays, it is revealed that the ubiquitous presence of these peptides among the plant kingdom play a noteworthy role in the innate immune system of plants. Plants that express defensins are highly resilient to fungal attacks and show augmented growth and development [6]. Active against a variety of human and fungal pathogens, these proteins have a potential use in therapeutics, medicines, as well as agriculture. As plant defensins play a very important role in defending plants against pathogenic microorganisms and other insects, they also interfere with the plant cells along with fungal cells. Some defensins can destroy microorganisms in 15–90 min by their disruptive actions on the cytoplasmic membrane. 

## 2. Origin of Defensins

At the start of the 1990s, numerous cationic plants with cysteine-abundance antimicrobial peptides were investigated. At first, plant defensins were reported in the seed products associated with whole wheat (*Triticum turgidum*) as well as barley (*Hordeum vulgare*) [7,8]. Initially, these peptides were categorized as a novel and detached member of the thionin family because of their resemblance in amino acid sequence, molecular weight, and the number of cysteines [7,8,9]. Nevertheless, subsequent research revealed significant variations within the arrangement of the disulfide bridge, showing no relationship between these two peptide families [10]. Broekaert et al. [11] renamed these types of peptides as plant defensins after evaluating their functional and structural similarities to formerly identified antimicrobial peptides (AMPs) found in mammals and insects. Defensins are structurally as well as functionally linked defense peptides that have been characterized in various eukaryotic microorganisms, as well as in mammals, plants, gulls, teleost species of fish, mollusks, insect pests, arachnidan, and crustaceans, in addition to fungi [11,12,13,14,15,16]. Phylogenetic studies indicated that these kinds of peptides share a common antecedent as tested among mollusk, arthropod, mammal, and bird defensins [17]. This prediction was usually reported because of the common interspecies conservation at different levels of peptide structures. The unique cysteine residue and the useful interspecies conservation affirmed that particular defensins present a common evolution. Depending on these types of results, the polyphyletic source associated with defensin peptides was uncertain. Through in silico studies, Zhu [18] revealed the existence of defensin-like peptides in *Anaeromyxobacter dehalogenans, Myxobacteria,* and *Stigmatella aurantiaca*. Despite the scarcity of details regarding antimicrobial activities, it is likely that these peptides characterize an antique method of defense inside prokaryotes that were transferred to the particular eukaryotic family during their progression [6]. In consonance with this particular perspective, six new groups of AMPs have been recognized in fungi. These families comprise 25 members containing defensins related to plant, insect, and invertebrate defensins [19]. This discovery provided insight into the resemblance of a bacterial peptide to two fungal defensin-like peptides, revealing that a bacterial antecedent contributed to these defensin molecules [19].

## 3. Structure of Defensins

**Figure 2:**
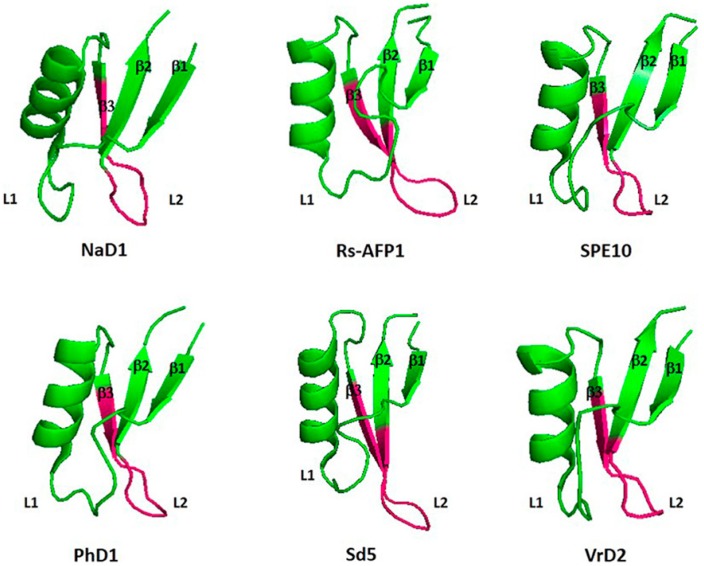
Three-dimensional structural conformation of six antifungal plant defensins (adopted from Lacerda et al. [3], an open-access article distributed under the terms of the Creative Commons Attribution License (CC BY)).

Plant defensins exhibit a highly conserved three-dimensional structural conformation comprised of one α-helix and three *β*-strands in antiparallel position. In addition, the arrangement of the amino acid sequence is also well conserved because of the occurrence of 6–8 cysteine residues constituting 3–4 disulfide interactions in the following order: Cys1–Cys8, Cys2–Cys5, Cys3–Cys6, and Cys4–Cys7 [20]. Nonetheless, plant defensins with five disulfide bridges have also been identified. The presence of an additional disulfide bond positioned after the α-helix and the primary *β*-sheet does not influence the typical three-dimensional structural organization of the defensins [21]. Moreover, the literature survey also revealed plant defensins with alternate structural organizations, including defensins from *Petunia hybrida* (PhD1 and PhD2), *Nicotiana alata* (NaD1), and ZmESR6 obtained from evolving maize kernels. These kinds of defensins comprise an additional acidic C-terminal pro-domain with still unknown functionalities. However, De Coninck and colleagues [22] reported its involvement in vacuolar targeting and circumventing damaging consequences caused by the basicity of the defensin. The sequence arrangement of plant defensins amino acids is not a conservative sequence, except the cysteines and a glycine located in the second *β*-sheet [23]. Figure 2 shows the three-dimensional structural conformation of six antifungal defensins from plants [3]. 

## 4. Mode of Action

**Figure 3:**
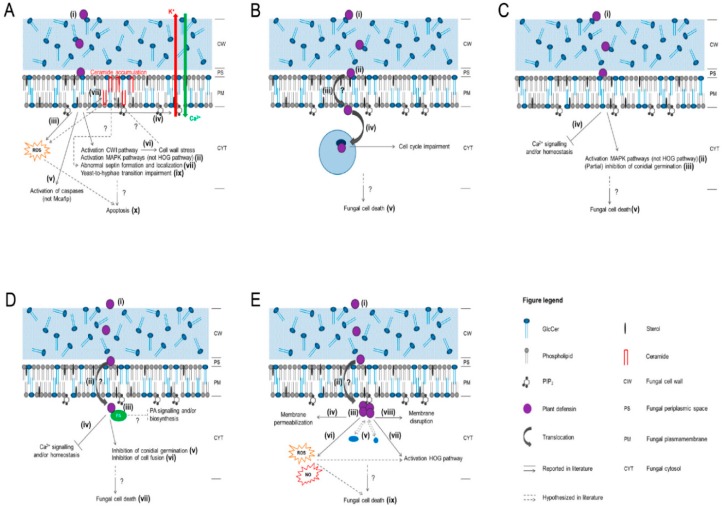
Schematic overview of the proposed mechanisms of action of the plant defensins. (**A**) RsAFP1 and RsAFP2; (**B**) Psd1; (**C**) MsDef1; (**D**) MtDef4; (**E**) NaD1. Reprinted from Vriens et al. [24], an open-access article distributed under the terms and conditions of the Creative Commons Attribution license (http://creativecommons.org/licenses/by/3.0/). Copyright (2014) the authors, Licensee MDPI, Basel, Switzerland.

A representation of the proposed action mechanisms of the plant defensins is shown in Figure 3 [24]. Indeed, the mechanism of antifungal defensins is most likely subject to electrostatic interactions in the middle of peptides and hyphal films, prompting a disturbance by a fast instigation of K^+^ efflux and Ca^+^ uptake and preventing parasitic growth. Notably, two major scientific hypotheses—the carpet model and the pore model—have been speculated to elucidate the action mechanism of antimicrobial defensin peptides. According to both models, defensins preferentially interrelate with negatively charged structures of pathogens’ cell membrane, resulting in increased membrane permeability and cell leakage followed by necrotic cell death. The carpet model explicates the pore formation of several peptides into the cell membrane, whereas the pore model demonstrates the formation of oligomers of those peptides, which then produce numerous pores into the membrane [3]. On the contrary, several reports have hypothesized an alternative mechanism of action of defensins without damaging the cell membrane of pathogens. In this hypothesis, these defense peptides are internalized into the intracellular environment, leading to elevated ion penetrability by reacting with the membrane phospholipids [25,26]. Therefore, they can also increase the generation of reactive oxygen species (ROS) and trigger apoptosis or intracellular programmed cell death [25,26]. The location of positively charged amino acids at loops or *β*-sheet regions has been reported to be useful for antifungal potentiality, suggesting that the interaction of positively charged Rs-AFP1 peptide with fungal pathogens might occur through electrostatic interfaces [27]. Some other reports focused on the structural assessments of plant defensins also recognized the significance of positively charged amino acid residues (located at the loop region) for antifungal activities, as well as working as a specificity factor against a range of pathogenic fungi [28]. Sagaram et al. [29] reported the presence of amino acid residues at the *γ*-core motif of MtDef4 as a crucial antifungal tool and specificity factor towards numerous pathogens. The mutagenesis studies of the RGFRRR region from MtDef4 revealed that the replacement of Arg and Phe at positions 4 and 3, respectively (positively charged hydrophobic residues), with Ala residues led to a significant deterioration of antifungal activity [29].

## 5. Role of Defensins in Plant Defense

**Figure 4:**
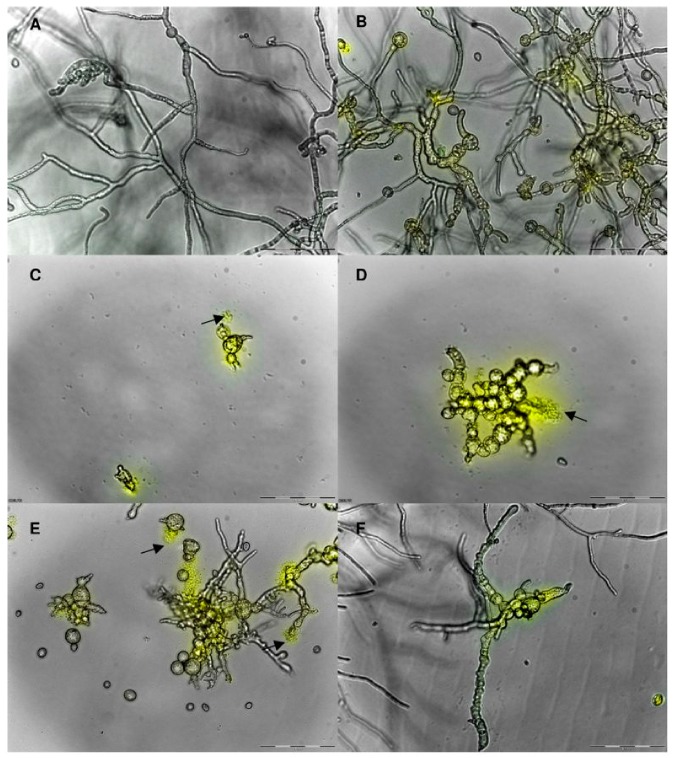
Combined overlay of the light microscopical analysis at 20× magnification and the cell permeabilization assay conducted on *B. cinerea* grown in the presence of Hc-AFPs for 48 h at 23 °C. (**A**) Control, (**B**) Hc-AFP1 25 μg/mL, (**C** and **D**) Hc-AFP2 15 μg/mL, (**E**) Hc-AFP3 25 μg/mL, (**F**) Hc-AFP4 18 μg/mL. The yellow fluorescence indicates a compromised membrane and the black arrows indicate structures that are leaking their cellular content into the surrounding medium. Adapted from De Beer and Vivier [31], an open-access article distributed under the terms of the Creative Commons Attribution License (http://creativecommons.org/licenses/by/2.0). Copyright (2011) the authors, licensee BioMed Central Ltd.

The protective role of defensin peptides in the protection of plants has been well described. Many reports have revealed that defensins are an essential component of the plant inherent immunity [30]. De Beer and Vivier [31] isolated four defensin genes (Hc-AFP1–4) with homology and clustered closest to defensins isolated from other Brassicaceae species. The same study also used propidium iodide assays to reveal the anti-fungal potential of all newly isolated defensin genes against *Botrytis cinerea*. A light microscopy analysis confirmed that the anti-fungal activity was related to an increase in membrane permeabilization (Figure 4) [31]. In summary, most of the plant defensins exhibited a constitutive expression pattern with upregulation following pathogens attacks, injuries, and abiotic stresses. Defensins are widely distributed and identified in flowers, tubers, leaves, pods, and seeds, where these peptides play a significant protective role during seed germination and seedling development [32]. Besides, plant defensins are also found in different tissues such as stomata, xylem, stomata, and parenchyma cells, and other peripheral regions [33]. Interestingly, plant defensins presented broad-spectrum antimicrobial activities, and some reports described the production of transgenic plants with the constitutive expression of foreign defensins. Therefore, these transgenic plants possess multiple biological potentialities, such as antibacterial, antifungal, and insecticidal activities, protein synthesis inhibition, inhibitors of digestive enzymes, and abiotic stress and heavy metal resistance [6,34]. Due to their potential biological activities, these defensins are categorized as promiscuous proteins. For instance, different homologous forms of a family of defensins isolated from *V. unguiculata* may present antibacterial and antifungal activities, as well as enzyme inhibition [35]. Though they display numerous biological activities, the antimicrobial role of plant defensins is predominantly noticed against a range of pathogenic fungi.

## 6. Peptides Involved in the Stress Response

**Figure 5:**
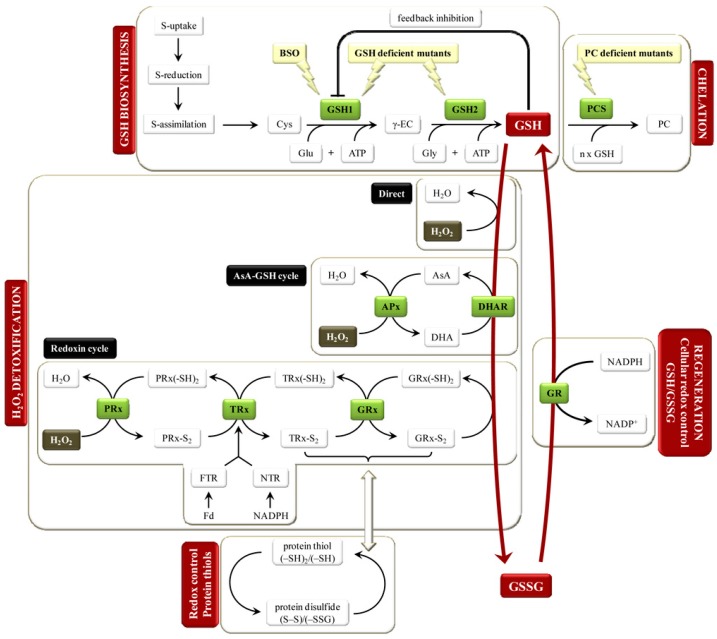
A schematic illustration of glutathione (GSH) biosynthesis and its involvement in chelation and redox control. Adapted from Jozefczak et al. [37], an open-access article distributed under the terms and conditions of the Creative Commons Attribution license (http://creativecommons.org/licenses/by/3.0/). Copyright (2012) the authors; licensee Molecular Diversity Preservation International, Basel, Switzerland.

Metal ions at higher concentrations are known to retard plant growth and development. Higher concentrations stimulate the generation of ROS such as free radicals, leading to oxidative stress. Plants exhibit defensive strategies such as cellular-free metal content (i.e., metal prohibition, cell wall binding, chelation, and sequestration), and governing cellular responses (i.e., anti-oxidative defense and the repair of stress-damaged proteins to cope with diverse types of these toxic metals) [36]. However, the synthesis of explicit chelators followed by metal complexes sequestration is of prime significance to restrict concentrations of free metals. As a key component of the metal-scavenging system, glutathione is a peptide that controls the metal ions uptake in response to ROS in plants due to its high affinity to metals [37]. The biosynthesis of glutathione (GSH) and its contribution in chelation–redox control are schematically shown in Figure 5 [37]. In addition, it acts as an important precursor of phytochelatins (PCs) that form complexes with heavy metals, which can then easily be accommodated into vacuoles. It has been observed that these PCs are effective in retaining high levels of metals in tobacco and other plants. These are also involved in the transport of metals. PCs are synthesized under specific conditions of plant growth and development. The activity of glutamylcysteine synthase, phytochelatin synthase, and serine acetyltransferase enzymes determine their synthesis and the binding capacity of metals to different sites [37].

## 7. Involvement of Peptides in Reproduction

SCR/SP11 (S locus cysteine-rich) is a peptide of 15 units. It consists of eight cysteine residues and its structure resembles that of defensins. Its structure is helpful in interaction with sigma kinase. LAT52, a member of this family, is important in developing a connection between stigma and pollen, which enhances hydration and the sprouting of the pollen tube. Another type of peptide, LTPs, were found to exhibit the same function in pollen growth when studied in *Arabidopsis thaliana*. These are slightly larger at 70 units. Therefore, these are not subjected to proteolysis and secreted like other peptides. In *Liliumlongi florum,* peptide SCA (stigma/style cysteine-rich adhesin) is involved in the attachment of pollens. This peptide works in association with chemocyanin and exhibits chemotropic behavior towards the pollen tube. It is a plant cyanin, which contains Cu as a binder. A defensin named LURE, which has been found to contain this cysteine, acts in defense and reproduction in *Torenia fournieri* L. Here, this defensin functions as a chemoattractant for pollens. In maize, it is secreted by synergid cells and helps in the release of sperms from pollens. LURE contains disulfide bonds that assure attachment of egg with sperm. It is actively released upon the approach of sperms in the ovary. ZmTLA1 is a peptide found in maize. Its nature is hydrophobic and it acts as a proteolipid. It is present in protoplast and actively takes part in the maturation of pollens [38].

## 8. Biological Functionalities of Plant Defensins

Different plant defensins possess multiple biological functionalities due to the huge variations in amino acid sequences on the surface loops. Notable functions include antibacterial activity, the inhibition of protein formation, α-amylase and trypsin enzyme interference, heavy metals resistance, and plant growth, development, and sexual reproduction [7,39,40,41,42]. Among these functions, antifungal activity is the most common function and is a well-characterized function of plant defensins.

### 8.1. Plant Defensins—Antimicrobial Activity

**Figure 6:**
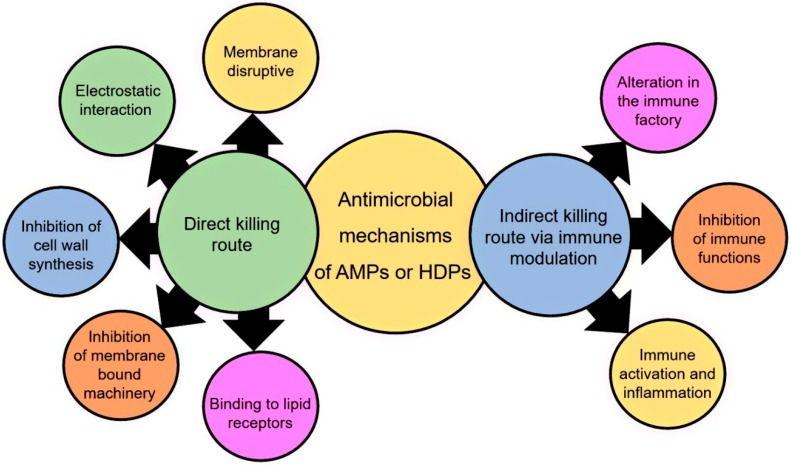
Potential antimicrobial mechanisms of plant defense-based antimicrobial peptides (AMPs) or host defense peptides HDPs.

In the early 1990s, Terras and colleagues [43] revealed that the antimicrobial activity of plant defensins was predominantly investigated against fungal pathogens. Nevertheless, some bacterial strains particularly belonging to the Gram-positive group were also detected to be suppressed by plant defensins, but the activity was less pronounced as compared to fungi. The growth of Gram-positive bacteria inhibited by plant defensins include *Bacillus subtilis*, *Bacillus cereus*, *Bacillus megaterium*, *Curtobacterium flaccumfaciens*, *Clavibacter michiganensis*, *Staphylococcus aureus*, *Staphylococcus epidermidis*, *Sarcina lutea*, and *Mycobacterium phlei*. Amongst the Gram-negative bacterial strains tried in inhibition bioassays were *Agrobacterium tumefaciens*, *Agrobacterium rhizogens*, *Agrobacterium radiobacter*, *Azospirillum brasilense*, *Alcaligenes eutrophus*, *Erwinia carotovora*, *Escherichia coli*, *Proteus vulgaris*, *Pseudomonas aeruginosa*, *Pseudomonas synrigae*, *Pseudomonas cichorii*, *Pseudomonas fluorescens*, *Pseudomonas lachrymans*, and *Salmonella typhimurium* [43,44,45,46,47,48,49,50]. Antibacterial activity is the most important characteristic of the vertebrate *trans*-defensins; however, it is less common in the *cis*-defensins family peptides from plants. In *trans*-defensins, disulfides orient in opposite directions and link to different secondary structure elements. Meanwhile, in *cis*-defensins, disulfides orient to the same cysteine-stabilized α-helix. Except for fabatins from the broad bean, *Vicia faba* has a profound inhibitory potential towards Gram-negative *Pseudomonas aeruginosa* and is moderately active against *Enterococcus hirae* and *Escherichia coli*. Notably, they have bacteria-specific activity, and thus exhibit no activity against *Candida albicans* or *Saccharomyces cerevisiae* [51]. In contrary, defensins from other *Fabaceae* members such as Ct-AMP1 from *Clitoria terna* and VaD1 from azuki bean are active against bacteria as well as fungal species [45]. As compared to lipid II binding by vertebrate defensins, plant defensins commonly carry out their antibacterial activity by binding to other lipids, i.e., phospholipids and fungus-specific sphingolipids [52,53]. Unlike bacteria, fungal pathogens are a more common risk faced by plants; this explains the prevalence of antifungal defensins over defensins with antibacterial activity. Several reports have shown the inhibition in growth of an array of fungal species by incubation with plant peptides. These strains and phytopathogens include *Aspergillus niger*, *Saccharomyces cerevisiae*, *Neurospora crassa*, *Alternaria solani*, *Alternaria brassicola*, *Cladosporium sphaerospermum*, *Fusarium oxysporum*, *Cladosporium colocasiae*, *Colletotrichum lindemuthianum*, *Fusarium decemcellulare*, *Fusarium culmorum*, *Fusarium graminearum*, *Fusarium verticillioides*, *Nectria haematococca*, *Penicillium expansum*, *Penicillium digitatum*, *Rhizoctonia solani*, *Septoria tritici*, *Trichoderma viride*, *Verticilium alboatrum*, and *Verticillium dahliae* [45,54,55,56,57,58,59,60]. The suppressive activity and required concentration of defensin for inhibition varies and depends on specific fungal pathogens and the plant defensin. Some potential antimicrobial mechanisms of plant defensin-based AMPs or host defense peptides (HDPs) are shown in Figure 6. 

### 8.2. Plant Defensins—Anticancer Activity 

Cancer is one of the prevalent causes of worldwide mortality, with approximately 8.2 million deaths in 2012 [61]. In spite of progress made in cancer treatment, conventional chemotherapy presents the serious disadvantage of broad-spectrum toxicity. The use of AMPs appeared as a unique and alternative family of anticancer agents to overcome the drawbacks of chemotherapeutic drugs [62,63]. Defensin-like peptides and plant defensins, in addition to their antimicrobial activities, also possess potential anticancer and cytotoxicity effects [63]. Wong and Ng [64] reported the first plant defensin, sesquin from *Vigna sesquipedalis*, which showed anticancer activity and repressed the growth of leukemia M1 and MCF-7 cell lines. Later on, the same research group found a limenin defensin from *Phaseolus limensis* that caused 30% and 60% proliferation inhibition of L1210 and M1 leukemia cells, respectively [65]. Lunatusin, another anticancer defensin obtained from *Phaseolus lunatus* seeds, suppressed the propagation of MCF-7 cancer cell lines. However, the cell-free inhibitory activity of lunatusin in the reticulocytes system of rabbit indicates its cytotoxicity towards normal cell types and tissues [66]. Subsequently, many reports have identified a number of different plant defensins with great potential to inhibit the multiplication of colon and breast cancer cell lines without exhibiting any cytotoxic effects on normal types. For example, Lin and colleagues [67] identified a defensin from *Phaseolus vulgaris* that potentially suppressed the growth of various cancer cells such as MCF-7, HepG2, HT-29, and Sila without affecting human erythrocytes or embryonic liver cells under the identical conditions. The proliferation of L1210 and HL60 cells was inhibited by a coccinin defensin peptide from *Phaseolus coccineus*, but it did not exhibit any cytotoxic influence on the propagation of mouse spleen cells [68]. Likewise, *Phaseolus coccineus*-derived phaseococcin possessed profound inhibitory activity against L1210 and HL60 cells without affecting the normal proliferation of rabbit reticulocytes or mouse splenocytes [69]. Without any effect on immortalized bovine endothelial cells, the complete inhibition of HeLa cells viability was achieved by 𝛾-thionin defensin from *Capsicum chinense* [70]. Generally, the mechanism of anticancer activity of plant defensins is poorly elucidated. Though a study by Lobo et al. [71] unveiled the only mechanism of action speculated to date, experimental studies are still necessary for further corroboration. Indeed, antimicrobial peptides have been found to possess an amphipathic three-dimensional structural organization, with one positively charged hydrophilic face and another hydrophobic portion of the molecule. Notably, these charged faces of the plant defensin molecules constitute an initial electrostatic binding with structures of opposite charge on the surface of the pathogenic microorganisms [34]. This speculation is substantiated by charged structures on the surface of microorganisms/mammalian cells and charged amino acids [72,73,74]. The hydrophobic portion following the initial binding is moved near the cell membrane, resulting in the lysis of the membrane. In contrast to normal cells, mammalian cancer cells exhibit a greater negative surface charge because of their high transmembrane potential and the aberrant expression of sialic acid. As a result, these cancer cells have an electrophoretic influence on antimicrobial peptides and thus attract them towards the membrane [75,76]. The interaction between defensing peptides and cancer cells presenting abnormal sphingolipids related to tumor development is another alternative mechanism of action, but lacks practical validation [34]. 

## 9. Concluding Remarks and Future Prospects

Increasing pathogen resistance to conventional antibiotics and inadequate health treatment options have intensified the development of new treatment approaches to overcome these challenges. In these scenarios, cationic plant peptides are very important for many biotechnological and medicinal purposes owing to their broad-spectrum biological activities. These plant defensins can also be produced in the eukaryotic host by heterologous expression due to non-toxic effects to mammalian cells. Notably, these defensin peptides from many plants such as *Abutilon indicum* can be used to treat many kinds of infectious diseases such as tuberculosis, piles, and liver disorders. Moreover, they can also be used to cure a variety of cancers. In addition, antifungal defensin-based agro-bioproducts are expected to be targeted as an essential means to improve crop productivity in the near future. Given the accelerated development of peptide libraries, bioinformatics, proteomics, and agriculture biotechnology strategies, these plant defensins could emerge as novel antimicrobial or anticancer drugs for a myriad of medical applications.
